# Histone H3K9 and H3K14 acetylation at the promoter of the *LGALS9* gene is associated with mRNA levels in cervical cancer cells

**DOI:** 10.1002/2211-5463.12973

**Published:** 2020-09-24

**Authors:** Erick Armenta‐Castro, Tania Reyes‐Vallejo, Daniel Máximo‐Sánchez, Irma Herrera‐Camacho, Gustavo López‐López, Sandra Reyes‐Carmona, Ileana Conde‐Rodríguez, Ivonne Ramírez‐Díaz, Adriana Aguilar‐Lemarroy, Luis Felipe Jave‐Suárez, Lorena Milflores‐Flores, Gerardo Santos‐Lopez, Julio Reyes‐Leyva, Verónica Vallejo‐Ruiz

**Affiliations:** ^1^ Laboratorio de Biología Molecular Centro de Investigación Biomédica de Oriente Instituto Mexicano del Seguro Social Metepec, Puebla México; ^2^ Posgrado en Ciencias Químicas Instituto de Ciencias Benemérita Universidad Autónoma de Puebla Puebla México; ^3^ Departamento de Ciencias Químico‐Biológicas Universidad de las Américas Puebla Puebla México; ^4^ Facultad de Ciencias Químicas Benemérita Universidad Autónoma de Puebla Puebla México; ^5^ Centro de Investigaciones en Ciencias Microbiológicas Instituto de Ciencias Benemérita Universidad Autónoma de Puebla Puebla México; ^6^ Centro de Investigación Biomédica de Occidente Instituto Mexicano del Seguro Social Guadalajara México; ^7^ Facultad de Ciencias Biológicas Benemérita Universidad Autónoma de Puebla Puebla México

**Keywords:** cervical cancer, galectin‐9, histone acetylation, *LGALS9*, mRNA variants, promoter methylation

## Abstract

Galectin‐9 levels have been reported to be altered in several cancer types, but the mechanism that regulates the expression of Galectin‐9 has not been clarified. Galectin‐9 is encoded by the *LGALS9* gene, which gives rise to eight mRNA variants. The aims of this study were: (a) to identify the mRNA variants of *LGALS9*, (b) to characterize CpG methylation and H3K9 and H3K14 histone acetylation at the promoter of the *LGALS9* gene, and (c) to characterize the relationship between these modifications and *LGALS9* expression level in cervical cancer cells. All mRNA variants were detected in HaCaT (nontumoural keratinocytes) and SiHa cells, and seven were observed in HeLa cells. The promoter region of *LGALS9* contains eight CpG dinucleotides. No hypermethylation pattern related to low *LGALS9* expression was identified in tumour cells. Chromatin immunoprecipitation analysis demonstrated higher acetylation of H3K9ac and H3K14ac in HaCaT cells, which was related to higher mRNA levels. The presence of the mRNA variants suggests that alternative splicing may regulate the expression of galectin‐9 isoforms. The results of this study suggest that histone acetylation, but not promoter CpG methylation, may be involved in the transcriptional regulation of the *LGALS9* gene.

## Graphical abstract

Altered expression of the *LGALS9* gene has been reported for several cancer types. This study showed that increased histone acetylation at the *LGALS9* promoter was associated with higher mRNA levels of *LGALS9*. In a nontumoral cell line, HaCaT, the *LGALS9* gene presented higher levels of histone acetylation than in cervical cancer cells. Our work suggests that epigenetic mechanisms may regulate the expression of the *LGALS9* gene during tumor transformation.

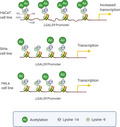

**Abbreviations**
CTs: threshold cycle
dNTPs: deoxynucleotide triphosphates
histone acetylation marks in Lys14: H3K14ac
histone acetylation marks in Lys9: H3K9ac
CC: cervical cancer
ChIP: chromatin immunoprecipitation
EPD: Eukaryotic Promoter Database
HPV: human papillomavirus
ICC: immunocytochemistry
IF: immunofluorescence assay
L: large
M: medium
qPCR: quantitative PCR
S: small
Tm: melting temperature

Cervical cancer (CC) is the second most lethal cancer among women in undeveloped countries [1]. The aetiological agent for this cancer is human papillomavirus (HPV), and persistent infection with some genotypes of HPV has been associated with the development of CC [2].

Galectin‐9 is a lectin that contains two carbohydrate binding domains joined by a peptide linker that recognize structures containing β‐galactoside [3]. This protein participates in different cellular functions, such as cell adhesion, apoptosis, migration and immune response, and serves as a signalling modulator [4].

During tumour transformation, galectin‐9 modifies its expression. Most of the studies in solid tumours suggest an inverse relation between galectin‐9 expression and cancer progression, including CC [5, 6, 7, 8]. Galectin‐9 is coded by the *LGALS9* gene, conformed by 11 exons. Eight mRNA variants have been reported for this gene, which result from alternative splicing and are named FL, D5, D5/6, D6, D10, D6/10, D5/10 and D5/6/10 according to the eliminated exon [4]. These variants encode different protein isoforms that are not well characterized, but some studies showed that they could play different roles [4, 9]. The best‐studied isoforms are the large (L), medium (M) and small (S) isoforms, which correspond to the mRNA isoforms FL, D5 and D5/6, respectively [4]. The mechanisms that regulate the expression of *LGALS9* have not been elucidated. Some studies report that the expression of *LGALS9* increases in the presence of interferon‐ɣ [10]. Epigenetic alterations play important roles in gene expression and may participate in the altered expression of *LGALS9* reported in cancer. Evidence indicates that epigenetic alterations are involved in various diseases, including cancer [11, 12, 13]. Epigenetic mechanisms, such as DNA methylation, and histone modifications, such as histone acetylation, are the most heavily studied epigenetic mechanisms due to their close relationship with gene expression levels. In cancer, promoter hypermethylation is related to the repression of tumour suppressor genes [14, 15]. Epigenetic alterations have been proposed as potential diagnostic biomarkers [16]. In premalignant cervical lesions and CC, a panel of methylated genes has been reported as possible biomarkers [17]. In contrast, histone acetylation is an activator modification that is mostly observed in active promoters and enhancers [18]. Histone acetylation marks in Lys9 (H3K9ac) and in Lys14 (H3K14ac) are related to the activation of gene expression [19, 20]. In cancer, hyperacetylation can be related to the expression of proto‐oncogenes, whereas hypoacetylation has been related to the silencing of tumour suppressor genes [21, 22].

In CC, alterations in enzymes participating in the regulation of the epigenome have been reported, affecting mechanisms such as DNA methylation and histone acetylation. These changes have been related to the oncoproteins E6 and E7 of high‐risk HPV [23, 24].

The aim of this study was to identify the mRNA variants of the *LGALS9* gene, to characterize CpG methylation and H3K9 and H3K14 histone acetylation at the promoter region of the *LGALS9* gene, and to determine their relationship with the expression level of this gene in CC cells.

## Materials and methods

### Cell culture

The human keratinocyte cell line (HaCaT) and CC cell lines SiHa and HeLa were cultured in Dulbecco's modified Eagle's medium (Sigma, St. Louis, MO, USA) with 10 mm Hepes, supplemented with 5% FBS, 100 U·mL^−1^ penicillin and 100 µg·mL^−1^ streptomycin (Sigma‐Aldrich; Merck KGaA, Darmstadt, Germany), and were maintained in an atmosphere with 5% CO_2_ at 37 °C. Subconfluent cells were harvested using a mixture of trypsin (0.025%) and EDTA (0.02%; Sigma) and washed with PBS.

### Identification of the mRNA variants of the *LGALS9* gene

RNA extraction from the cell lines was performed using the NucleoSpin RNA Mini kit (Macherey‐Nagel, Düren, Germany) following the manufacturer's instructions. The integrity of the RNA was verified by agarose gel electrophoresis, and the purity and concentration were determined by spectrophotometry.

cDNA synthesis was performed using Revert Aid H Minus First Strand cDNA (Thermo Fisher Scientific, Inc., Waltham, MA, USA). Two micrograms of RNA, 1 µL of oligo dT primers, 4 µL of reaction buffer, 1 µL of nuclease inhibitor, 2 µL of deoxynucleotide triphosphates (dNTPs) (10 mm), 1 µL of RevertAid and water were added to a final volume of 20 µL. The reaction was incubated at 42 °C for 60 min followed by incubation at 70 °C for 5 min. This reaction was performed for all of the cell lines and was used for the detection of the mRNA variants and for the ALL primers that amplify all the variants.

For PCR, PCR Master Mix (Promega, Madison, WI, USA) was used, and the reaction was performed in a Veriti thermocycler (Applied Biosystems, Thermo Fisher Scientific, Inc., Foster, CA, USA) using 5 µL of 2X PCR Master Mix, 0.5 µL of forward (10 µm) and reverse (10 µm) primers, and 1 µL of cDNA and water to a final volume of 10 µL. The programme conditions for the mRNA variants were as follows: 95 °C for 10 min followed by 40 cycles of 95 °C for 10 s, 61 °C for 30 s and 72 °C for 30 s. For the ALL primers, the conditions were the same, except that the melting temperature (*T*_m)_ was 58 °C. The sequences and size of the amplification products are shown in Table 1. The amplification products were electrophoresed in agarose gel at 2.5%.

**Table 1:** Sequences of oligonucleotides used to amplify all and each mRNA variant, the size of the product amplified and the name of the mRNA variant.

Primer pair	Forward (5′–3′)	Reverse (5′–3′)	Product length (bp)
LGALS9 ALL	CTTTCATCACCACCATTCTG	CTCTGAGCACTGGGCAGG	81
LGALS9 LC	GCAGACAAAAACCTCCCG	CCCAGAGCACAGGTTGATG	254
LGALS9 D5	ATCAGCTTCCAGCCTCCC	CCCAGAGCACAGGTTGATG	255
LGALS9 D6	GCAGACAAAAAACCCAGACA	CCCAGAGCACAGGTTGATG	218
LGALS9 D10	GCAGACAAAAACCTCCCG	TTCACACAAGATCCACACCTCT	247
LGALS9 D5/6	CTACATCAGCTTCCAGACCCA	CCCAGAGCACAGGTTGATG	223
LGALS9 D5/10	ATCAGCTTCCAGCCTCCC	TTCACACAAGATCCACACCTCT	248
LGALS9 D6/10	GCAGACAAAAAACCCAGACA	TTCACACAAGATCCACACCTCT	211
LGALS9 D5/6/10	CTACATCAGCTTCCAGACCCA	TTCACACAAGATCCACACCTCT	216
HPRT	CCTGGCGTCGTGATTAGTGATGAT	CGAGCAAGACGTTCAGTCCTGTC	147

### Determination of mRNA levels of *LGALS9* by quantitative RT‐PCR

The optimal amplification efficiencies of the *LGALS9* gene and the endogenous *HPRT* gene were determined for the ALL primers that amplify all of the mRNA variants and for the FL and D5 mRNA variants. To this end, a concentration curve was performed from   20 to 0.02 ng·µL^−1^using the cDNA from the HaCaT cell line. A melting curve was performed to confirm that unspecific products were not obtained during the amplification. Each reaction was performed in a final volume of 10 µL, including 5 µL of 2X SYBR Green/Rox quantitative PCR (qPCR) Master Mix (Thermo Fisher Scientific, Inc.), 1 µL of forward primer (10 µm), 1 µL of reverse primer (10 µm) and 1 µL of cDNA (20 ng) from the HaCaT, SiHa and HeLa cell lines. The reactions were performed in the Step One Real‐Time PCR System (Applied Biosystems, Thermo Fisher Scientific, Inc.).

The reactions were performed in triplicate in three different assays using the Step One Real‐Time PCR System (Applied Biosystems, Thermo Fisher Scientific, Inc.). The programme conditions for the mRNA variants were as follows: 95 °C for 10 min followed by 40 cycles of 95 °C for 10 s, 61 °C for 30 s and 70 °C for 30 s. For the ALL primers, the conditions were the same, but the *T*_m_ was 51 °C. The expression levels were determined using the $2^{\Delta \Delta C_{t}}$ method.

### Expression of galectin‐9 by immunocytochemistry and immunofluorescence

To determine the expression of galectin‐9 in HaCaT and cervical cell lines, we performed an immunocytochemistry (ICC) and immunofluorescence assay (IF). A protocol suggested by Abcam for ICC and IF was followed. HaCaT, SiHa and HeLa cell lines were cultured in an eight‐well Chamber Slide™ system (C7182; Merck) and fixed with 100% methanol for 5 min at room temperature. Nonspecific antibody binding was blocked with 1% BSA in PBS for 1 h. Then cells were incubated overnight at 4 °C with an anti‐galectin‐9 antibody (ab69639; Abcam) diluted 1 : 400 with 1% BSA in PBS. Next, cells were incubated for 1 h at room temperature with anti‐rabbit IgG (Alexa Fluor® 488) (ab150077; Abcam) diluted 1 : 1000 in 1% BSA in PBS. Nuclei staining was performed with 0.1 µg·mL^−1^ DAPI Stain (4083; Cell Signaling Technology, Danvers, MA, USA). An isotype IgG control was applied as a negative control test. Fluorescence intensity was determined using the program zen 2.6 Lite from Zeiss (Oberkochen, Germany). The ratios of cytoplasmic and nuclear level fluorescence were also determined for each cell line.

### Identification of CpG dinucleotides at the promoter region of *LGALS9* by *in silico* analysis

The promoter region for the *LGALS9* gene was identified by *in silico* analysis using the Eukaryotic Promoter Database (EPD). The identification of CpG islands was performed with the DataBase of CpG Islands and Analytical Tools [25].

### Analysis of CpG methylation

DNA extraction from the cell lines was performed using the Wizard® Genomic DNA Purification kit (Promega). The integrity of the DNA was verified by agarose gel electrophoresis.

For the methylation assay, 2 µg of DNA was treated with sodium bisulphite using the EZ DNA Methylation‐Gold™ kit (Zymo Research, Irvine, CA, USA). The absence of unconverted DNA was checked by PCR amplification using LGALS9_F1 and LGALS9_R1 (Table 2). Several fragments containing the dinucleotide CpG were amplified using the primers shown in Table 2.

**Table 2:** Sequences of oligonucleotides used for the amplification and sequencing of fragments of the promoter of the *LGALS9* gene to identify methylation in the CpG dinucleotides. The sequences of oligonucleotides used in the qPCR after ChIP assay are also listed.

Oligonucleotide name	Application	Sequence (5ʹ–3ʹ)
LGALS9_F1	DNA methylation	CCAGCCTCTCAAAGACTCCTTCC
LGALS9_R1		CAGCTTTACTCATCATTGCCCCAG
BS_LGALS9_F2		GTGTAGAAAATAAGGATGGAGAAGA
BS_LGALS9_R2		CACAACTAATTCATAACCACCTAAAC
BS_LGALS9_F3		GTTTAGGTGGTTATGAATTAGTTGTG
BS_LGALS9_R3		CCTAAACATACTTAACCACAAAATCC
BS_LGALS9_F4		GGATTTTGTGGTTAAGTATGTTTAGG
BS_LGALS9_R4		CCAATAACCTTTCCCTCCTCCCTCT
BS_LGALS9_F5		AGAGGGAGGAGGGAAAGGTTATTGG
BS_LGALS9_R5		CTAAAAACCACTAAAAACCATCTCTCCAC
ChIP_LGALS9_F6	ChIP	CATAAAGTTCAGGCGGCCACG
ChIP_LGALS9_R6		CTGTGTGTCCTAAGCATGCTTGG

The PCR analysis was performed in a final volume of 50 µL using the PCR Master Mix Kit (Promega), 100 ng of DNA and a final concentration of forward and reverse oligonucleotides of 1 µm. The amplification product was purified with the QIAquick® PCR Purification kit (Qiagen, Inc., Valencia, CA, USA). The PCR products were sequenced by the Sanger method in LANGEBIO (Laboratorio Nacional de Genómica para la Biodiversidad from CINVESTAV‐IPN). The oligonucleotides used were PG9Me_F5, PG9Me_R6, PG9Me_F7 and PG9Me_R8 according to the amplification product. The sequence analysis was performed using the program Chromas, and multiple alignments were performed employing the tool Clustal Omega from EMBL‐EBI (https://www.ebi.ac.uk/Tools/msa/clustalo/) considering as a consensus sequences those obtained with the EPD program at the Gene database National Center for Biotechnology Information.

### Chromatin immunoprecipitation and qPCR

To evaluate acetylation of histone H3 in Lys9 (H3K9ac) and Lys14 (H3K14ac) in the *LGALS9* promoter in HaCaT, SiHa and HeLa cell lines, we performed a chromatin immunoprecipitation (ChIP) assay using the ChIP plus Sonication kit (56383; Cell Signaling Technology). All of the cell lines were fixed with 1% formaldehyde, and chromatin was sheared by sonication into 500‐bp DNA–protein fragments with eight cycles of 15 seconds ON/60 seconds OFF. Anti‐H3K9ac (9649; Cell Signaling Technology), anti‐H3K14ac (7627; Cell Signaling Technology), positive control anti‐histone H3 (4620; Cell Signaling Technology) and mock control normal rabbit IgG antibody (2729; Cell Signaling Technology) were used according to the manufacturer's protocol. A 2% input was recovered from each cell line before immunoprecipitation. For quantification, a qPCR assay was performed. For each reaction, 1 µL of DNA from the HaCaT, SiHa and HeLa cell lines was used in a final volume of 10 µL, including 5 µL of 2X SYBR Green/Rox qPCR Master Mix (Thermo Fisher Scientific, Inc.), 1 µL of 10 µm forward primer and 1 µL of 10 µm reverse primer. The reactions were performed in the Step One Real‐Time PCR System (Applied Biosystems, Thermo Fisher Scientific, Inc.). The programme conditions were as follows: 95 °C for 10 min, 40 cycles of 95 °C for 30 s, 60 °C for 30 s and 72 °C for 30 s, and 72 °C for 5 min as a final extension. H3K9ac and H3K14ac levels were determined using the percent input method.

### Statistical analysis

A one‐way ANOVA followed by Tukey’s posttest was performed to determine the differences in the expression level using the graphpad program (version 7; GraphPad Software, Inc., La Jolla, CA, USA). Significant differences were considered as *P* < 0.05.

## Results

### Quantification of *LGALS9* mRNA levels

**Fig. 1:**
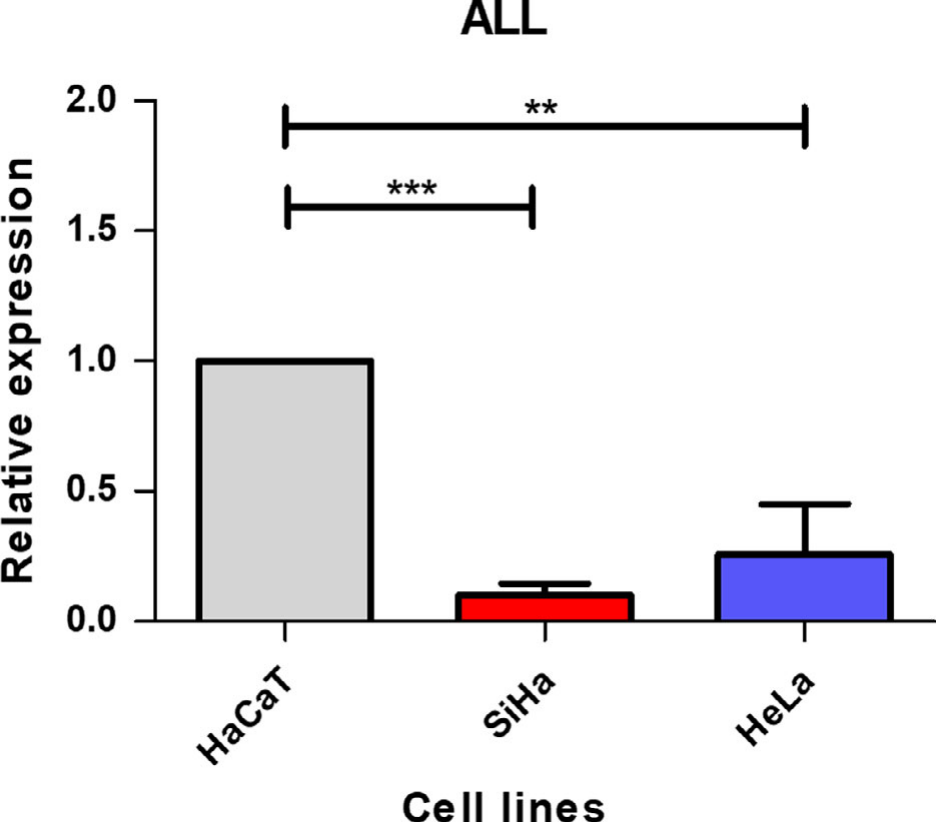
Relative expression of the *LGALS9* gene in the HaCaT, SiHa and HeLa cell lines. The expression level was determined with quantitative RT‐PCR using the comparative CT method. The HaCaT cell line (nontumoural) showed higher expression. Error bars represent the standard error of the mean for three independent experiments. **P < 0.01, ***P < 0.001, one‐way ANOVA, Tukey's *post hoc* test.

The quantification of mRNA levels was determined using the ALL primers that amplify all of the mRNA variants, and the results showed higher expression in the HaCaT cell line than in the CC cell lines (Fig. 1).

### Identification of mRNA variants of *LGALS9* in HaCaT and CC cell lines

**Fig. 2:**
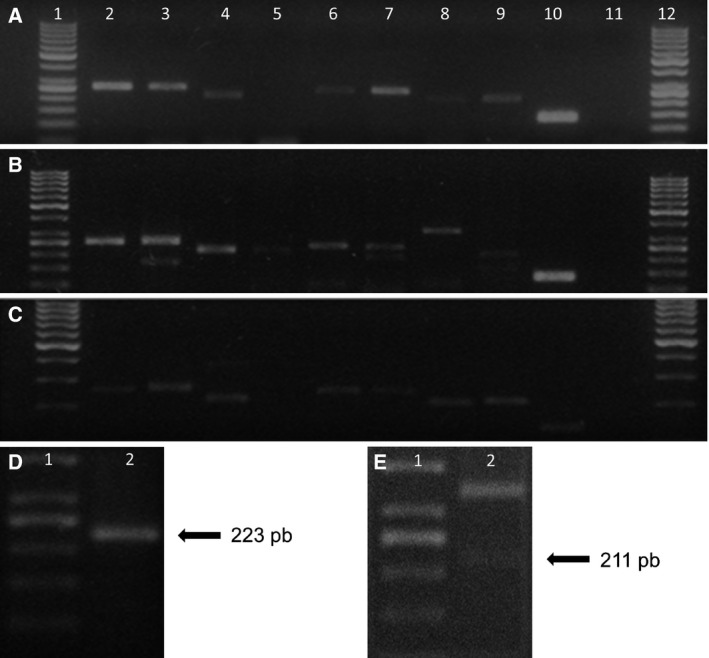
Electrophoresis in agarose gel of the PCR products of the mRNA variants of the *LGALS9* gene. (A) HaCaT cell line. (B) SiHa cell line. (C) HeLa cells. The image shows in each line the mRNA variant detected. Lane 1: molecular weight; lane 2: FL variant; lane 3: D5 variant; lane 4: D6 variant; lane 5: D5/D6 variant; lane 6: D10 variant; lane 7: D5/D10 variant; lane 8: D6/D10 variant; lane 9: D5/D6/D10 variant; lane 10: HPRT control; lane 11: negative; lane 12: molecular weight. (D) The amplification product for the mRNA variant D5/D6 in the HaCaT cell line. (E) The mRNA variant D6/10 in the SiHa cell line; however, the corresponding band was very faint.

The mRNA variants in the HaCaT (Fig. 2A), SiHa (Fig. 2B) and HeLa (Fig. 2C) cells were detected in an agarose gel electrophoresis of the amplified PCR products. The D5/D6 product was not observed for HaCaT and HeLa cells, but the presence of this variant was confirmed in a posterior PCR analysis with a higher concentration of cDNA for HaCaT cells (Fig. 2D). For the SiHa cell line, the mRNA variant D6/D10 was not observed in the gel; nevertheless, a higher size band was observed. The presence of the D6/D10 variant was also confirmed in a posterior PCR analysis with a higher concentration of cDNA (Fig. 2E). We detected the presence of the eight mRNA variants in HaCaT and SiHa cells, and only the D5/D6 variant could not be detected in HeLa cells.

### Quantification of FL and D5 mRNA variants in HaCaT and cervical cell lines

**Fig. 3:**
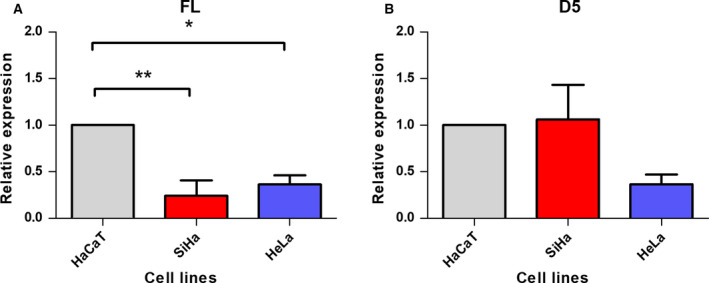
Expression levels of the FL and D5 transcripts in HaCaT, SiHa and HeLa cell lines. The expression level was determined with RT‐PCR using the comparative CT method. (A) The results showed that the HaCaT cell line presents higher expression levels for the FL variants. (B) For the D5 mRNA variant, there were no statistically significant differences. Error bars represent the standard error of the mean for three independent experiments. *P < 0.05, **P < 0.01, one‐way ANOVA, Tukey’s *post hoc* test.

We performed a quantification analysis by qPCR only for the variants FL and D5. The other variants (D6, D5/D6, D10, D5/10, D6/10 and D5/6/10) were not quantified, because the expression levels of these variants were extremely low and the products were detected at very late threshold cycle (CTs). The analysis showed a higher expression of FL in HaCaT cells with respect to CC cells (Fig. 3A). For the D5 variant, no significant differences were observed (Fig. 3B).

### Expression of galectin‐9 in HaCaT and CC cell lines

**Fig. 4:**
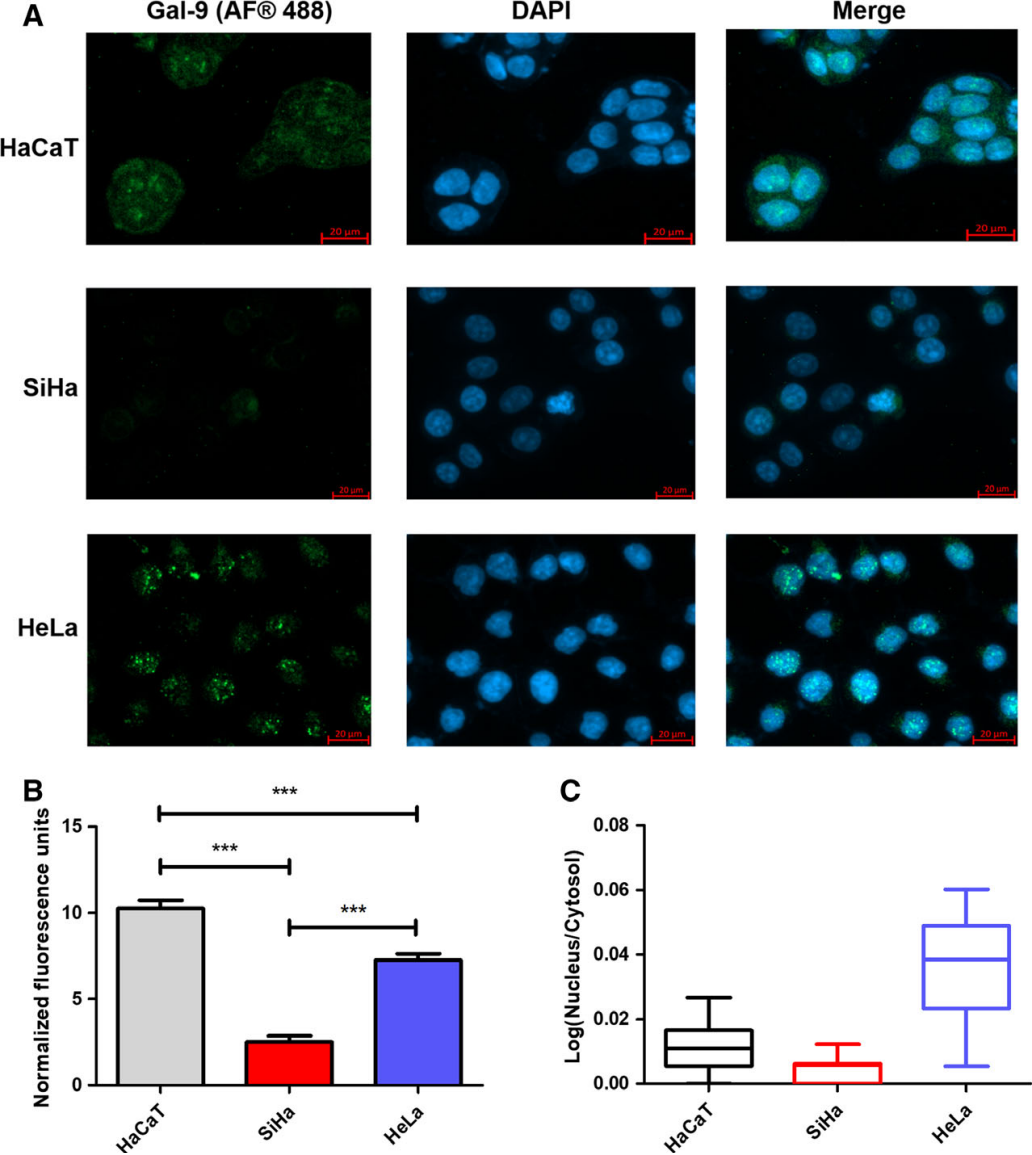
Galectin‐9 detection in HaCaT, SiHa and HeLa cell lines by IF. (A) Galectin‐9 expression (green, secondary labelled with Alexa Fluor® 488) in HaCaT, SiHa and HeLa cell lines, and nuclear staining with DAPI (blue). In HaCaT cells, the presence of galectin‐9 was observed in the cytoplasm and the nucleus, and in HeLa cells, the presence of galectin‐9 was observed predominantly in the nucleus. In SiHa cells, the signal of galectin‐9 was very faint. Scale bars: 20 µm. (B) Fluorescence intensity was plotted for each cell line. For this, 20 cells were quantified in five different areas, and the error bars represent the standard error of the mean. The highest expression of galectin‐9 was detected in the HaCaT cell line. ***P < 0.0001, one‐way ANOVA, Tukey’s *post hoc* test. (C) Nuclear/cytosol fluorescence signal ratios are shown for each cell line. Nuclear galectin‐9 is higher in HeLa than in HaCaT and SiHa cells.

Expression of galectin‐9 in HaCaT and cervical cell lines was evaluated by ICC‐IF (Fig. 4A). In HaCaT cells galectin‐9 was localized in the cytoplasm and the nucleus, in SiHa cells galectin‐9 expression was very faint, and in HeLa cells galectin‐9 was observed predominantly in the nucleus (Fig. 4B,C). A higher galectin‐9 staining was observed in the HaCaT cell line.

### DNA methylation of CpG sites in the *LGALS9* promoter

**Fig. 5:**
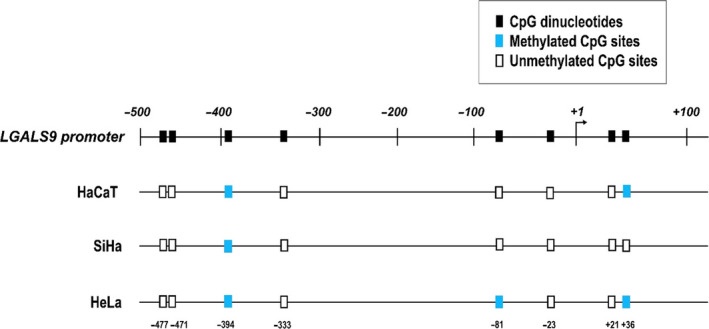
CpG dinucleotides and DNA methylation pattern in the *LGALS9* promoter. The first line is a representation of the promoter region considered for the methylation analysis, where black boxes indicate CpG dinucleotide locations. The following lines represent the DNA methylation status in the *LGALS9* promoter in HaCaT, SiHa and HeLa: methylated CpG sites (blue boxes) and unmethylated CpG sites (white boxes).

The *in silico* analysis of the *LGALS9* gene using the EPD programme showed a possible *LGALS9* core promoter at position −50 and +10 with respect to the start site of transcription; however, other regulatory elements are present around this region and conform to the promoter region. Thus, we examined a region between −500 and +100 to identify the potential methylation sites. DataBase of CpG Islands and Analytical Tools showed a lack of CpG islands in the *LGALS9* promoter region, but we identified eight CpG dinucleotides where DNA methylation can occur. These sites were located at positions −477, −471, −394, −333, −81, −23, +21 and +36 (Fig. 5).

The methylation analysis showed a conserved methylated CpG in the analyzed cell lines at position −394, another methylated CpG was detected in HaCaT and HeLa cells at position +36, and a third CpG methylated site was detected in HeLa cells at position −81 (Fig. 4). No specific DNA methylation pattern was detected for the differentiation between tumoural and nontumoural cell lines.

### Histone H3K9 and H3K14 acetylation in the *LGALS9* promoter

**Fig. 6:**
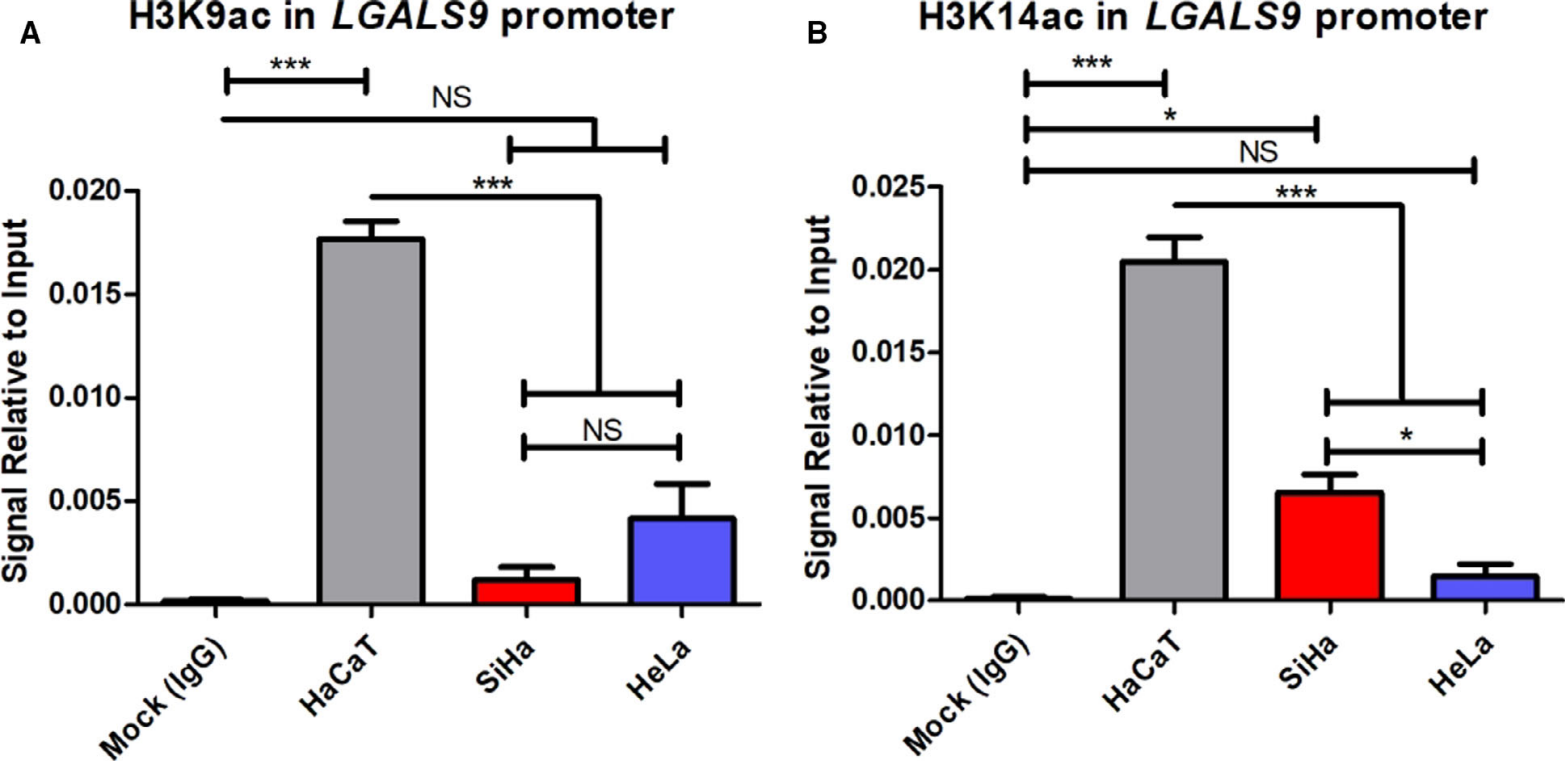
ChIP‐qPCR of H3K9ac and H3K14ac in the *LGALS9* promoter. We report that H3K9ac (A) and H3K14ac (B) are decreased in tumoural cell lines (SiHa and HeLa) compared with the nontumoural cell line (HaCaT). Error bars represent the standard error of the mean for three independent experiments. ***P < 0.0001, *P < 0.005, one‐way ANOVA, Tukey’s *post hoc* test. NS, not significant.

To determine whether acetylation is involved in *LGALS9* gene expression, we performed a ChIP assay with anti‐H3K9ac and anti‐H3K14ac antibodies. Next, we quantified these marks with qPCR amplification. In Fig. 6A, we observe that H3K9ac was decreased in SiHa and HeLa cells compared with HaCaT cells. Simultaneously, the mock control and the signal detected in SiHa and HeLa cells showed no significant differences, which confirms the low level of H3K9ac in tumoural cell lines. In contrast, we observed that H3K14ac is also decreased in SiHa and HeLa cell lines compared with HaCaT (Fig. 6B), but SiHa has higher H3K14ac levels compared with HeLa, which showed no significant differences compared with the mock control.

## Discussion

In our study, higher mRNA levels of the *LGALS9* gene were detected in HaCaT nontumoural cells, whereas SiHa and HeLa CC cells, which are derived from cervical squamous cell carcinoma and adenocarcinoma, respectively, exhibited low expression of the gene. The results agree with the down‐regulation of galectin‐9 reported in biopsies of CC [6, 8]. The expression of galectin‐9 in cervical adenocarcinoma has not been reported, but it could also be down‐regulated, as observed in the adenocarcinoma cell line HeLa. It has been proposed that galectin‐9 could play an antitumoural role, inducing the apoptosis of tumour cells and inhibiting cell invasion [26, 27, 28].

Eight mRNA variants resulting from alternative splicing have been reported for the *LGALS9* gene. These variants encoded different protein isoforms. The mRNA variants that do not contain the exons 5 and 6 coded isoforms with a shorter linker domain, and the mRNA variants with exon 10 deleted give rise to proteins with the carboxyl carbohydrate binding domain truncated that are not secreted; therefore, its functions are at the intracellular level [4].

This study is the first that identifies the mRNA variants of *LGALS9* in CC cells. For SiHa cells, we detected all of the mRNA variants, and for HeLa cells, we did not detect the presence of the D5/D6 variant. The regulation of the expression of the different variants could play a role in the cellular functions of galectin‐9, as previously reported for L and M, which play different roles [4, 29]. Galectin‐9 isoforms M and S decreased the expression of E‐selectin in colon carcinoma cells, in contrast with galectin‐9L, which increased its expression [9]. Heusschen *et al*. reported that galectin‐9M can modulate proliferation and migration, but its function depends on the cell type, its concentration and location. In endothelial cells, five mRNA variants were identified (FL, D5, D5/D6, D5/D6 and D10) [29]. Another study reported the presence of six variants corresponding to FL, D5, D5/D6, D5/D6, D10 and D6 in the placenta, and the decrease in the variant D5/D6 was associated with spontaneous abortion [4]. These results highlight the importance of the regulation of alternative splicing of LGALS9 mRNA and help to characterize the functions of the protein isoforms. In our study, the mRNA variants with higher expression correspond to FL and D5, which encode galectin‐9L and galectin‐9M, respectively. The expression level for the FL variant is higher in the nontumoural cell line compared with the tumoural cell lines, and for D5, we observed similar expression levels between the tumoural and nontumoural cells, suggesting different roles of galectin‐9 in relation to the expression levels of the variants. The expression of galectin‐9 in the analyzed cell lines showed differences in its subcellular localization: in HaCaT cells, galectin‐9 was observed in the cytoplasm and the nucleus, whereas in HeLa cells, its expression was observed predominantly in the nucleus as protein aggregates. Changes in the subcellular localization have been reported for some galectins in cancer. Expression of galectin‐7 in normal oesophageal epithelial tissues was observed primarily in the nucleus, but in the oesophageal epithelial cancer tissue, galectin‐7 was observed both in the nucleus and in the cytoplasm [30]. In tongue carcinomas biopsies, the subcellular localization of galectin‐3 is different from that of the normal mucosa; in tumour tissue, galectin‐3 was observed predominantly in the cytoplasm of the cells, whereas in the normal tissue, it was observed in the nucleus [31]. The roles of the galectins in cellular processes differ in relation to its subcellular localization [32].

The analysis of *LGALS9* promoter methylation showed that there is no relation with the expression level of the gene in HaCaT, SiHa and HeLa cells, and a hypermethylation status was not found in the cervical cell lines, although low expression levels were observed for the gene. According to the BiSearch Web Server (http://bisearch.enzim.hu/), the promoter region analyzed in this study was located between positions 27630516 and 2763102. However, Nair *et al*. [33] reported epigenetic analysis in a region of the *LGALS9* gene located between 27646372 and 27646761 in colorectal cancer; although the analyzed region is different, they found no changes in DNA methylation or on histone repressive marks. In contrast, Zhang et al. [34] studied CpG methylation by methylation‐specific PCR in CC samples and found differences; nevertheless, the analyzed region was located between 27628429 and 27628426. A more extensive analysis is warranted to determine whether promoter methylation could regulate the expression of *LGALS9*.

The analysis of H3K9 and H3K14 acetylation showed that these markers were higher in HaCaT cells than in the tumoural cell lines analyzed in this study. The relationship between H3K9ac and gene expression has been previously reported [35, 36], and H3K14ac is also involved in this process [37]. The elevated presence of these marks adjacent to the promoter suggests a possible role in *LGALS9* gene expression. Because these marks are important for gene transcription [19], the reduced abundance of these marks in tumoural cell lines could be involved in *LGALS9* gene expression. Nevertheless, it is important to complete epigenetic studies analyzing other active and repressive marks, which could provide more information regarding the complex epigenetic mechanism regulating the expression of this gene.

## Conclusions

Our results showed that the epigenetic marks H3K9ac and H3K14ac, but not CpG methylation, at the promoter region are related to the mRNA expression levels of *LGALS9*. Furthermore, the identification of mRNA variants in HaCaT, SiHa and HeLa cells suggests that the regulation of alternative splicing and the expression of protein isoforms could modify the functions of galectin‐9. The subcellular location of galectin‐9 differs between the nontumoral HaCaT and HeLa cells.

## Conflict of interest

The authors declare no conflict of interest.

## Author contributions

EA‐C, VV‐R and JR‐L conceived and designed the project. EAC performed the methodology. EA‐C, TR‐V and DM‐S acquired the data. TR‐V, IR‐D, IC‐R, SR‐C, IH‐C, DM‐S, GS‐L, VV‐R and GL‐L analyzed and interpreted the data. EA‐C, AA‐L, LFJ‐S, LM‐F and GS‐L performed the *in silico* analysis. EA‐C, TR‐V, JR‐L and VV‐R wrote the paper. IH‐C, GL‐L and SR‐C provided supervision. All of the authors contributed to the revision of the manuscript.

## Data Availability

All data generated or analyzed during this study are included in this article.
